# Intelligent Volume-Assured Pressure Support vs. Spontaneous/Timed Mode as a Weaning Strategy in COPD—Evaluation of a New Ventilation Strategy

**DOI:** 10.3390/arm90040037

**Published:** 2022-07-28

**Authors:** Pradipta Bhakta, Mohanchandra Mandal, Prosenjit Mukherjee, Brian O’Brien, Antonio M. Esquinas

**Affiliations:** 1 Department of Anaesthesiology and Intensive Care, Hull University Teaching Hospital NHS Trust, Hull HU3 2JZ, East Yorkshire, UK; 2 Institute of Post Graduate Medical Education and Research, Kolkata 700020, West Bengal, India; drmcmandal@gmail.com (M.M.); docposhu@gmail.com (P.M.); 3 Cork University Hospital Group, Wilton, T12 DC4A Cork, Ireland; drbobrien@hotmail.com; 4 Intensive Care Unit, Hospital Morales, 30008 Murcia, Spain; antmesquinas@gmail.com

We thank Salama S et al. for their evaluation of noninvasive positive-pressure ventilation (NPPV) strategies in patients with chronic obstructive pulmonary disease (COPD) and who are suffering an acute exacerbation thereof [1]. We would like to query some choices for and aspects of the methodology of the study.

Firstly, higher HACOR (a composite of heart rate, acidosis, consciousness, oxygenation, and respiratory rate) scores, as well as the trend in the scores, better predicts NPPV failure when assessed within 24 h [2,3]. We wondered whether the authors had considered this for predicting NPPV failure?

Secondly, they evaluated patient comfort and quality of mask-fitting using a visual analogue scale to determine overall acceptance, as well as tolerance to NPPV, using the iVAPS mode. iVAPS is a novel dual-hybrid mode of NPPV which offers continuous automatic breath-to-breath adjustment of the level of pressure support to achieve a desired target alveolar ventilation [4]. Hence, it may be better than the S/T mode of NPPV during sleep in COPD patients due to the changing respiratory mechanics and variable ventilatory demands [5,6]. Ekkernkamp E et al. found a favourable sleep score with the use of the iVAPS mode compared to the high-intensity assist–control NPPV mode in COPD patients [5]. iVAPS was also found to improve the apnoea–hypopnoea and arousal indices in patients with chronic respiratory failure [7]. The use of iVAPS is known to improve health-related quality of life after recovery from acute illness [5,8]. It is not clear whether Salama S et al. observed any improvement in sleep quality with the use of iVAPS in their patients.

Thirdly, only the height of patients was used to predict physiological dead space which was used to calculate target alveolar ventilation in the iVAPS group. Ideally, body weight and height, and hence, body mass index, might have been used. They are known to affect functional residual capacity and respiratory mechanics [9].

Fourthly, although the groups were comparable regarding the duration of NPPV use and intensive care unit (ICU) stay, it is not clear what criteria the authors used to guide the discontinuation of NPPV or discharge from the ICU. This warrants clarification.

Finally, the authors used the ratio of partial pressure of arterial oxygen tension and the fraction of inspired oxygen concentration (PaO_2_/FiO_2_) as criteria for detecting NPPV failure and the subsequent need for re-intubation, as well as the initiation of invasive mechanical ventilation (IMV). The respiratory rate oxygenation (ROX) index, on the other hand, can evaluate the combined impact of oxygenation (PaO_2_/FiO_2_) as well as respiration [10]. ROX monitoring could have been used during weaning from the NPPV when they used RSBI for detecting the NPPV weaning failure.

In the study in question, the authors did not find any differences in the outcomes or satisfaction scores between the iVAPS and S/T modes of NPPV in acute exacerbations of COPD during weaning from IMV. We think clarity on the above-mentioned points might aid the overall evaluation of the outcome of this study. Further well-designed, higher-powered studies are perhaps warranted to identify the subset of patients who might benefit most from the use of this innovative NPPV mode.
